# Secondary metastasis in the lymph node of the bowel invaded by colon cancer: a report of three cases

**DOI:** 10.1186/s12957-016-1026-y

**Published:** 2016-10-26

**Authors:** Aki Takiyama, Hiroaki Nozawa, Soichiro Ishihara, Hirotoshi Takiyama, Koji Murono, Koji Yasuda, Kensuke Otani, Takeshi Nishikawa, Toshiaki Tanaka, Tomomichi Kiyomatsu, Kazushige Kawai, Keisuke Hata, Toshiaki Watanabe

**Affiliations:** Department of Surgical Oncology, The University of Tokyo, 7-3-1 Hongo, Bunkyo-ku, Tokyo, 113-8655 Japan

**Keywords:** Colorectal cancer, Locally invasive cancer, Lymph node metastasis, Adjacent organ

## Abstract

**Background:**

Secondary metastasis to regional lymph nodes for adjacent bowel invaded by colorectal cancers (CRCs) has not been extensively reviewed. We herein present three such cases.

**Case presentation:**

The first case is a cancer involving the cecum and sigmoid colon, and its primary site could not be determined even by pathological evaluation. Nodal involvement was revealed both in the mesocolon of the cecum and sigmoid. The second and third cases are a sigmoid colon cancer invading the jejunum and an ascending colon cancer invading the jejunum, respectively. These patients harbored secondary metastases to lymph nodes draining from the invaded small bowel segments. In spite of complete resection, all three patients metachronously developed liver metastases or recurrent disseminated nodules in the pelvis and subsequently died.

**Conclusions:**

In cases of CRC invading another bowel segment, bowel resection with regional lymphadenectomy for both involved segments should be considered to achieve complete resection. However, the radical surgery did not necessarily provide a long-term survival.

## Background

Complete surgical resection provides the best possibility of long-term survival for patients with colorectal cancer (CRC). Invasion to the adjacent organs occurs in 10–20 % of CRC cases, which requires multivisceral resection (MVR) for radical operation [1–3]. Most studies of MVR in CRC have focused on the clinicopathological characteristics of primary CRC, postoperative complications, and/or long-term prognosis [1–12]. However, there have been few reports describing regional lymph node metastasis in adjacent organs invaded by CRC. We report three such cases and discuss the treatment strategy of locally invasive CRC.

## Case presentation

### Case 1

**Fig. 1:**
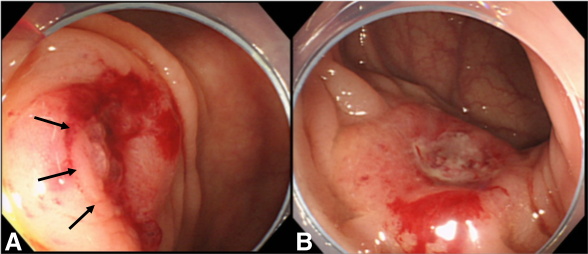
**a** Case 1: tumor occupying the appendiceal orifice (*arrows*). **b** Case 1: type 2 tumor at the sigmoid colon

**Fig. 2:**
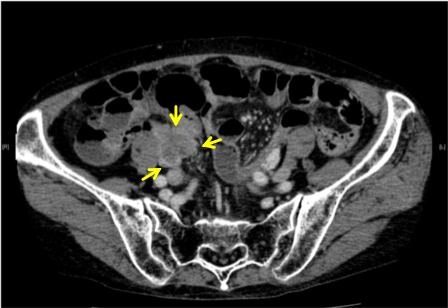
Case 1: 3-cm mass involving the sigmoid colon, cecum, and terminal ileum (*arrows*)

An 80-year-old woman underwent total colonoscopy due to a positive fecal occult blood test. A tumor at the appendiceal orifice (Fig. 1a) and a type 2 tumor occupying a quarter of the perimeter wall of the sigmoid colon were identified (Fig. 1b). Biopsied specimens of both tumors showed adenocarcinoma. CT scans showed a 3-cm mass involving the sigmoid colon, cecum, and terminal ileum (Fig. 2). At operation, a tumor involving the appendix and right ovary, as well as five disseminated nodules at Douglas’ pouch, was identified. We performed MVR including complete removal of the visible nodules, and histological examination of the resected specimens could not determine the primary site of the adenocarcinoma, which was 2.5 × 3 cm in size and invading multiple organs. Metastases were revealed in the para-colic lymph nodes of both the cecum and sigmoid colon. Although oral 5-fluorouracil (5-FU) was administered as adjuvant chemotherapy for 6 months, the patient showed recurrent disseminated nodules in the pelvis and died 31 months after the operation.

### Case 2

**Fig. 3:**
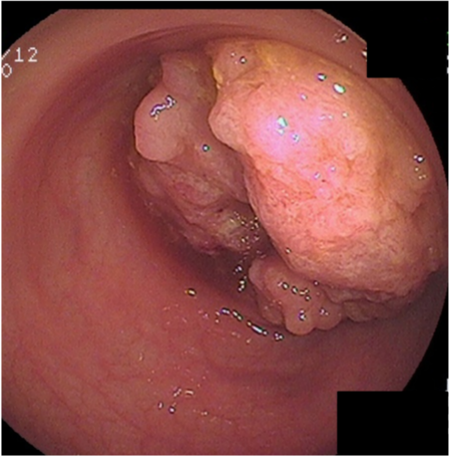
Case 2: colonoscopy revealed an obstructive sigmoid colon cancer

**Fig. 4:**
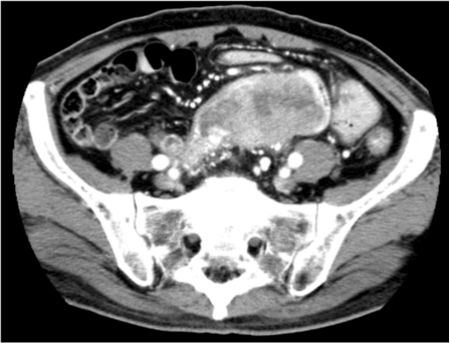
Case 2: abdominal CT scan showed a large mass in the sigmoid colon

A 79-year-old man with constipation and weight loss underwent colonoscopy and was diagnosed with obstructive sigmoid colon cancer (Fig. 3). CT scans suggested a large tumor in the sigmoid colon involving the jejunum (Fig. 4). At operation, a 10-cm tumor in the sigmoid colon was identified directly invading the jejunum. Additionally, enlarged lymph nodes in the mesenterium of the involved segment of the jejunum were found. Resection of the sigmoid colon as well as the jejunal segment was performed, with lymph node retrieval. Pathological examination confirmed that the colon cancer penetrated through the jejunal wall, causing ulcer formation. No nodal metastasis was identified at the sigmoid colon, whereas one lymph node was metastasis-positive at the jejunum.

Liver metastases appeared 5 months after the operation. Although the patient received immunotherapy, his condition gradually deteriorated. Twenty-four months later, 5-FU-based chemotherapy was initiated; he died 48 months after the operation.

### Case 3

**Fig. 5:**
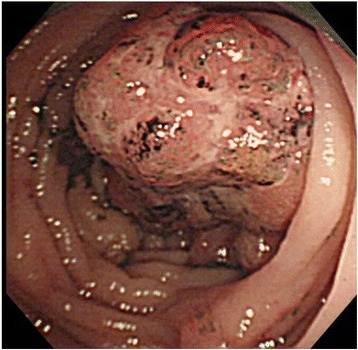
Case 3: circumferential tumor in the ascending colon

**Fig. 6:**
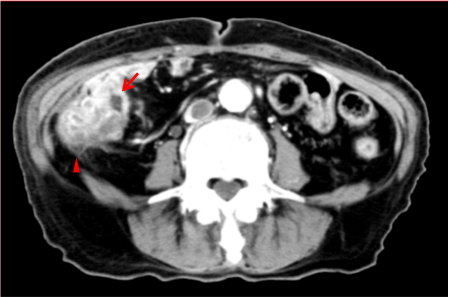
Case 3: ascending colon cancer (*arrowhead*) invading the adjacent small bowel (*arrow*)

A 76-year-old man was hospitalized due to weight loss, appetite loss, progressive anemia, edema of the leg, and dehydration. Colonoscopy showed a circumferential tumor in the ascending colon (Fig. 5). Abdominal CT scans revealed an ascending colon tumor, and its boundary against the adjacent small bowel was unclear (Fig. 6). Right hemicolectomy, partial resection of the jejunum and partial abdominal wall resection were performed. Pathological examination revealed that the 9-cm tumor in the ascending colon invaded directly into the jejunum. Two lymph nodes were positive for metastasis in the mesenterium of the small intestine. Recurrent lesions were found in the liver 67 months after the operation. He refused hepatectomy and died shortly after.

### Discussion

Advanced CRCs occasionally invade adjacent organs, such as the bladder, ureter, small or large intestine, liver, uterus, ovary, vagina, prostate, seminal vesicle, and abdominal wall [1, 4, 5, 7, 8, 11, 12]. Among these, the bladder, reproductive organs, and small bowel are frequently resected in clinical practice [1, 3–6, 8, 11], and MVR has been reported to afford long-term survival [1–8, 11, 12].

There have been only a few reports regarding lymph node metastasis of the invaded organ. Ueno et al. [13] reported that of 783 cases of curatively resected colon cancer, 90 (11.5 %) required combined resection of the adjacent organs, including 22 cases of small bowel resection. They reported three cases with lymph nodes metastasis in the mesenterium of the small intestine; the primary colon cancer had invaded the mucosa of the small bowel in all cases. More recently, a case of transverse colon cancer invading the stomach with further metastasis to a node along the lesser curvature was reported [14].

Other studies, including the present cases, have reported the possibility of secondary metastases to the regional lymph nodes of invaded organs [13, 14]. Thus, retrieval of the draining lymph nodes of the invaded organs should be considered for radical surgery. On the other hand, there have been no reports in the literature of nodal involvement of the uterus, ovary, or bladder invaded by CRC. Thus, further investigation is required to determine whether radical lymphadenectomy should be applied for all invaded organs.

In the present report, all three patients died of recurrence in spite of complete resection. Therefore, adjuvant therapy should be considered to achieve better prognosis for such aggressive CRCs. Due to the paucity of this disease entity, further investigation and accumulation of patients is required.

## Conclusions

In cases of CRC invading another bowel segment, we should consider dissection of draining lymph nodes of the invaded bowel when they are suggestive of metastasis. Moreover, when determination of the primary site is difficult, bowel resection with regional lymphadenectomy for both segments should be considered to obtain complete resection.

## Abbreviations

5-FU: 5-Fluorouracil
CRC: Colorectal cancer
CT: Computed tomography
MVR: Multivisceral resection
